# Impact of aerobic exercise type on blood flow, muscle energy metabolism, and mitochondrial biogenesis in experimental lower extremity artery disease

**DOI:** 10.1038/s41598-020-70961-8

**Published:** 2020-08-20

**Authors:** Maxime Pellegrin, Karima Bouzourène, Jean-François Aubert, Christelle Bielmann, Rolf Gruetter, Nathalie Rosenblatt-Velin, Carole Poitry-Yamate, Lucia Mazzolai

**Affiliations:** 1 Division of Angiology, Heart and Vessel Department, University Hospital of Lausanne, Lausanne, Switzerland; 2 Center for Biomedical Imaging (CIBM), Ecole Polytechnique Fédérale de Lausanne (EPFL), Lausanne, Switzerland

**Keywords:** Cardiovascular models, Peripheral vascular disease

## Abstract

Exercise training (ET) is recommended for lower extremity artery disease (LEAD) management. However, there is still little information on the hemodynamic and metabolic adaptations by skeletal muscle with ET. We examined whether hindlimb perfusion/vascularization and muscle energy metabolism are altered differently by three types of aerobic ET. ApoE^−/−^ mice with LEAD were assigned to one of four groups for 4 weeks: sedentary (SED), forced treadmill running (FTR), voluntary wheel running (VWR), or forced swimming (FS). Voluntary exercise capacity was improved and equally as efficient with FTR and VWR, but remained unchanged with FS. Neither ischemic hindlimb perfusion and oxygenation, nor arteriolar density and mRNA expression of arteriogenic-related genes differed between groups. ^18^FDG PET imaging revealed no difference in the steady-state levels of phosphorylated ^18^FDG in ischemic and non-ischemic hindlimb muscle between groups, nor was glycogen content or mRNA and protein expression of glucose metabolism-related genes in ischemic muscle modified. mRNA (but not protein) expression of lipid metabolism-related genes was upregulated across all exercise groups, particularly by non-ischemic muscle. Markers of mitochondrial content (mitochondrial DNA content and citrate synthase activity) as well as mRNA expression of mitochondrial biogenesis-related genes in muscle were not increased with ET. Contrary to FTR and VWR, swimming was ineffective in improving voluntary exercise capacity. The underlying hindlimb hemodynamics or muscle energy metabolism are unable to explain the benefits of running exercise.

## Introduction

Lower extremity artery disease (LEAD) is a frequent arterial pathology affecting over 200 million people worldwide^1^. It consists in reduced lower limb blood flow and perfusion, secondary to atherosclerotic plaque stenosis or occlusion of lower limbs arteries. The most typical clinical presentation of LEAD in symptomatic patients is intermittent claudication (IC), which is characterized by ischemic muscular cramping in the legs due to inadequate blood flow delivery to exercising muscles. Consequently, LEAD patients with IC present with diminished walking capacity and lower limb function, in addition to muscle atrophy, resulting in diminished quality of life. Interventions aiming at improving walking performance and preventing functional lower limb decline are essential components of the clinical management of LEAD and IC^2,3^.

According to current international guidelines, the first-line conservative treatment modality for improving walking capacity in IC patients consists in pharmacotherapy and exercise training (ET)^2,3^. There is considerable evidence supporting the benefits of ET on walking capacity in LEAD patients. For example, a recent systemic review and meta-analysis demonstrated that ET improves pain-free walking distance and maximal walking distance, by respectively, 82 and 120 m in IC patients^4^. Structured walking ET (i.e. supervised exercise program and structured community- or home-based exercise program) is the main documented and prescribed exercise therapy for LEAD patients with IC towards improving walking distance; and it is more effective than unstructured, unsupervised walking (consisting of encouraging patients to walk more)^5^. Other alternative types (modes) of aerobic ET might, however, be equally beneficial as walking^6,7^. For example, swimming is a well-known exercise modality providing cardiovascular benefits^8^, and has been shown to be the second most preferred mode of exercise after walking in IC patients^9^. Despite its potential clinical relevance, the effect of swimming in LEAD has not been investigated so far^9^.

Although the efficiency of ET in improving walking capacity has been widely demonstrated in IC patients, the underlying mechanisms remain incompletely understood. Some biological mechanisms have been postulated, including those related to hemodynamic changes such as increased collateral circulation and/or improved calf muscle blood flow^10,11^. Other potential mechanisms may rely on intrinsic factors in the skeletal muscle, such as improved muscle energetics or increased mitochondrial biogenesis^10,11^. However, there is still little experimental evidence demonstrating such ET-mediated adaptations specifically in LEAD. Furthermore, it remains unknown whether ET type affects those mechanisms differently.

Therefore, using a LEAD and IC mouse model, the aim of the present study was to evaluate what effect aerobic ET type (forced running, voluntary running, and forced swimming) has on voluntary exercise capacity, hindlimb perfusion/vascularization and energy metabolism of muscle. We hypothesized that: (1) swimming is equally as effective as running in improving voluntary exercise capacity; and (2) ET would improve hindlimb blood flow and/or muscle energy metabolism, but to different degrees, depending on exercise training type.

## Results

### Characterization of the LEAD mouse model

ApoE^−/−^ mice underwent right iliac artery ligation, and at one week post-surgery, they were randomized into four groups for the reminder of the 5 weeks study: sedentary (SED), forced treadmill running (FTR), voluntary wheel running (VWR), or forced swimming (FS), as detailed in “Methods”.

Subsequent to iliac artery ligation, hindlimb tissue perfusion significantly decreased in all mice (laser Doppler imaging: 31.4 ± 1.8% at 1 week post-surgery vs. 99.9 ± 1.8% at pre-surgery, p < 0.0001, n = 33). In SED mice, hindlimb tissue perfusion remained low (68.2 ± 4.3%, p < 0.0001 vs. pre-surgery, n = 9) at 5 weeks post-surgery. Limb tissue oxygenation (TcPO_2_) similarly decreased in all mice, from 82.0 ± 4.7 mmHg at pre-surgery to 9.9 ± 1.9 mmHg at 1 week post-surgery (p < 0.0001, n = 51). In SED mice, TcPO_2_ remained low (38.8 ± 7.2 mmHg, p < 0.0001, n = 11) at 5 weeks post-surgery.

Relative to pre-surgery assessment, total running distance during 24 h (24 h-TRD), an indicator of voluntary exercise capacity, decreased by 47.8% in all mice at 1 week post-surgery (n = 38, p < 0.0001); it is noteworthy that a 55.2% reduction in 24 h-TRD was recorded 5 weeks post-surgery in the SED mice (n = 10, p < 0.0001). These results demonstrate the effectiveness of *chronic*, iliac artery ligation in establishing a model of chronic hindlimb ischemia and impaired walking capacity as previously described^12^.

At baseline, i.e. 1 week post-surgery, no significant difference in body weight (BW) between groups was observed (data not shown). However, at the end of the study, BW significantly decreased in FTR (p < 0.05) and VWR (p < 0.01) trained mice, but remained unchanged in mice of the FS group, when compared to the mice of the SED group (Table 1). Total plasma cholesterol levels were not significantly different between any of the four groups at the study endpoint (Table 1). As shown in Table 1, only the FS group was associated with a significantly increased ischemic lower limb muscle fiber area (p < 0.01 vs. SED). No significant differences were observed between groups for muscle fiber area in the non-ischemic lower limb muscle (Table 1). The number of regenerating fibers in ischemic lower limb muscle did not significantly differ between groups (Table 1).

**Table 1:** General characteristics of ApoE^−/−^ mice with LEAD at the study endpoint.

Group	Body weight (g)	Total cholesterol (g/L)	Ischemic gastrocnemius muscle fiber area (μm^2^)	Non-ischemic gastrocnemius muscle fiber area (μm^2^)	Ischemic gastrocnemius muscle regenerated fibers (% of total fibers)
SED	31.1 ± 0.5	4.57 ± 1.07	1,287 ± 186	2,228 ± 313	17.1 ± 5.4
FTR	29.3 ± 0.6^#^	4.90 ± 0.64	2024 ± 129^##^	3,084 ± 593	32.8 ± 10.2
VWR	28.6 ± 0.4^##^	5.12 ± 0.54	1,466 ± 90	3,697 ± 705	22.6 ± 9.7
FS	30.5 ± 0.4	7.01 ± 1.77	1,341 ± 91	4,641 ± 1,029	23.6 ± 10.2

Data are mean ± SEM (n = 16–21 animals per group for body weight; n = 6 animals per group for total cholesterol; n = 6–10 animals per group for gastrocnemius muscle fiber area and fiber regeneration).

Data were analyzed using one-way ANOVA with Bonferroni’s post-hoc test: ^#^P < 0.05, ^##^P < 0.01 vs. SED.

### Characteristics of exercise training protocols

Over the 4-week course of exercise training post-surgery, running distance by mice of the FTR and VWR groups was evaluated per exercise session, by week, over a duration of 4 weeks. In the FTR group, the mean total running distance was 23.2 ± 2.5 km, with an average of 1.25 ± 0.06 km per exercise session (n = 19). In the VWR protocol, mice covered 164.9 ± 12.3 km in total, averaging 6.14 ± 0.31 km per day (n = 19). While running distance in the FTR group was significantly higher at weeks 3 and 4, than at weeks 1 and 2, post-surgery (Supplementary Table 1), running distance increased at week 2 in the VWR group, and remained stable at week 3 and 4 post-surgery (Supplementary Table 1). LEAD ApoE^−/−^ mice in the FS group swam on average a total of 1,050 min (n = 17).

### Voluntary exercise capacity following exercise training

**Figure 1:**
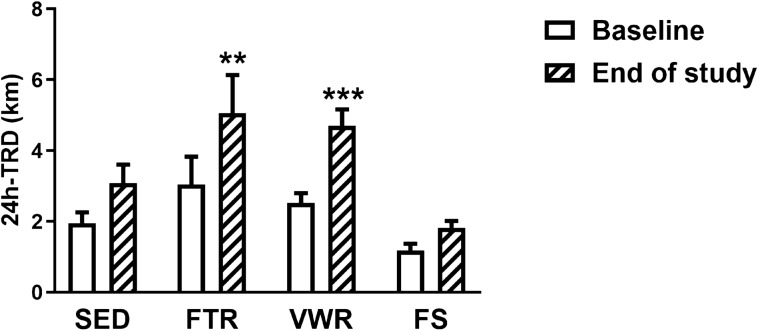
Effect of aerobic exercise training type on voluntary exercise capacity in ApoE^−/−^ mice with LEAD. Quantification of total running distance during 24 h (24 h-TRD) at baseline and at the study endpoint. Data represent mean ± SEM (n = 10 in SED; n = 8 in FTR, n = 10 in VWR, and n = 10 in FS). Data were analyzed using two-way repeated measures ANOVA with Bonferroni’s post-hoc test: **P < 0.01, ***P < 0.001 vs. baseline. In this and the following figures: *SED* sedentary controls, *FTR* forced treadmill running,* VWR* voluntary wheel running, *FS *forced swimming.

The effect of each exercise training regimen on the animal’s voluntary exercise capacity was assessed by measuring the total running distance over a 24 h period (24 h-TRD) at 2 time points: at baseline and at the end of the study. As shown in Fig. 1, there was no significant difference in 24 h-TRD between groups at baseline (1.96 ± 0.30 m, SED; 3.04 ± 0.79 m, FTR; 2.52 ± 0.27 m, VWR; 1.17 ± 0.19 m, FS). However, at the end of the study, the voluntary exercise capacity of mice of the FTR group significantly improved, relative to FTR baseline values (24 h-TRD + 93%, p < 0.01). A comparable improvement was found in the VWR group (24 h-TRD + 86%, p < 0.001), but no significant improvements were observed in the FS and SED mice. On the basis of these results, we concluded that FTR and VWR are equally effective in improving voluntary capacity exercise performance, compared to mice of the forced swimming and sedentary groups.

### Hindlimb tissue perfusion, oxygenation, and vascularization following exercise training

**Figure 2:**
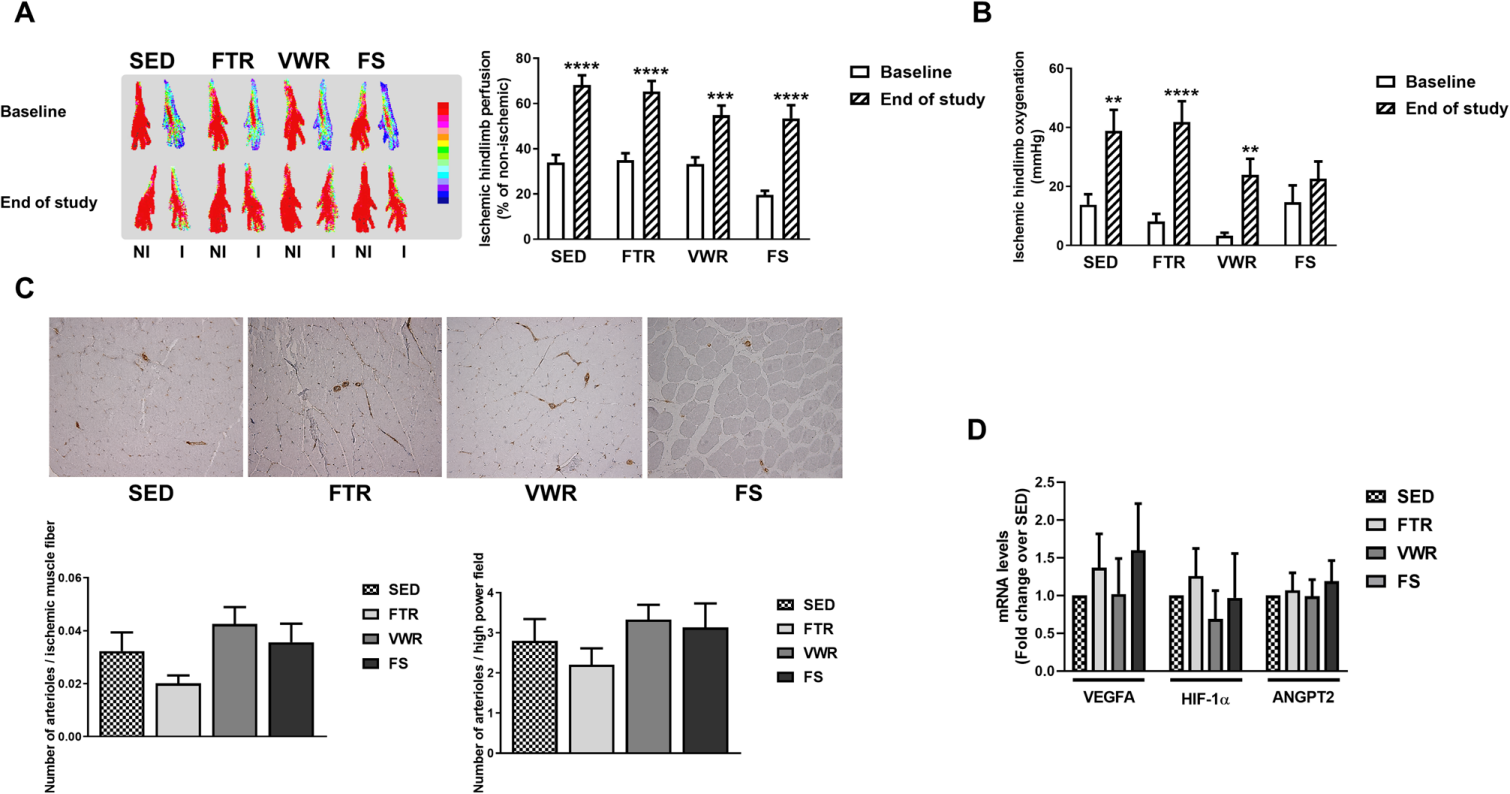
Effect of aerobic exercise training type on ischemic limb vascularization in ApoE^−/−^ mice with LEAD. **(A)** Left panel, Representative laser Doppler images of ischemic (I, right) and contralateral non-ischemic (NI, left) lower hindlimbs paws at baseline and at the study endpoint. The color scale ranges from blue (low perfusion) to red (high perfusion). Right panel, Quantification of ischemic hindlimb perfusion expressed as percentage of non-ischemic hindlimb perfusion. Data represent mean ± SEM (n = 9 in SED; n = 8 in FTR, n = 10 in VWR, and n = 6 in FS). **(B)** Quantification of ischemic hindlimb oxygenation using TcPO_2_ measurement (in mmHg) at baseline and at the study endpoint. Data represent mean ± SEM (n = 11 in SED; n = 15 in FTR, n = 12 in VWR, and n = 13 in FS). **(C)** Top panel, Representative photomicrographs of ischemic muscles immunostained with anti-α-SMA monoclonal antibody (magnification × 20). Bottom panel, Quantification of arteriolar density in ischemic gastrocnemius muscle at the study endpoint, expressed as the number of α-SMA-positive arterioles per muscle fiber and per high power field. Data represent mean ± SEM (n = 10 in SED; n = 8 in FTR, n = 9 in VWR, and n = 5 in FS). **(D)** mRNA expression of angiogenic/arteriogenic-related genes VEGFA, HIF-1α, and ANG2 in ischemic gastrocnemius muscle, as measured by quantitative real-time PCR at the study endpoint. Results in exercised groups were expressed as an x-fold change relative to SED, set at 1 (n = 9 in SED; n = 8 in FTR, n = 10 in VWR, and n = 6 in FS). Data were analyzed using two-way repeated measures ANOVA with Bonferroni’s post-hoc test (hindlimb perfusion and oxygenation data) or a one-way ANOVA with Dunnett’s post-hoc test (quantitative real-time PCR data): **P < 0.01, ***P < 0.001, ****P < 0.0001 vs. baseline.

We then addressed the question whether an improvement in voluntary endurance exercise performance, i.e. 24 h-RD might be associated with a change in hemodynamic parameters. The results are shown in Fig. 2A,B. At baseline, perfusion and oxygenation of ischemic hindlimbs were not significantly different between the SED, FTR, VWR and FS groups.

However, with the exception of FS oxygenation, ischemic hindlimb perfusion and oxygenation by the end of the study increased from baseline in all exercised groups of mice, as well as that in SED mice (Fig. 2A, B) (for perfusion by group: 65.3 ± 4.7%, FTR; 54.8 ± 4.3%, VWR; 53.4 ± 5.9%, FS; 68.2 ± 4.3%, SED. For oxygenation by group: 41.9 ± 7.1 mmHg, FTR; 23.9 ± 5.5 mmHg, VWR; 22.7 ± 5.8 mmHg, FS; 38.8 ± 7.2 mmHg, SED). The finding that this trend extended to the SED group of mice is attributed to, spontaneous recovery of perfusion and oxygenation of ischemic hindlimb muscle as previously reported^12^.

At the study endpoint, arteriolar density was estimated in ischemic hindlimb muscle as a function of ET type. The results are summarized in the histogram of Fig. 2C. Consistent with the perfusion and oxygenation findings, there was no significant difference in the number of arterioles per ischemic muscle fiber and per high power field between the exercised and SED groups. Arteriolar density was also estimated in non-ischemic muscle, and no significant difference was observed between the groups (Supplementary Fig. 1).

Lastly, mRNA expression of pro-angiogenic/arteriogenic vascular endothelial growth factor A (VEGFA), hypoxia inducible factor 1α (HIF-1α), and angiopoietin 2 (ANGPT2) did not significantly differ between groups (Fig. 2D). Moreover, changes in protein level of VEGFA between exercised and SED groups were not significant (data not shown).

These results led us to conclude that exercise training does not potentiate blood flow recovery in our LEAD mouse model.

### Glucose metabolism in hindlimb muscle following exercise training

**Figure 3:**
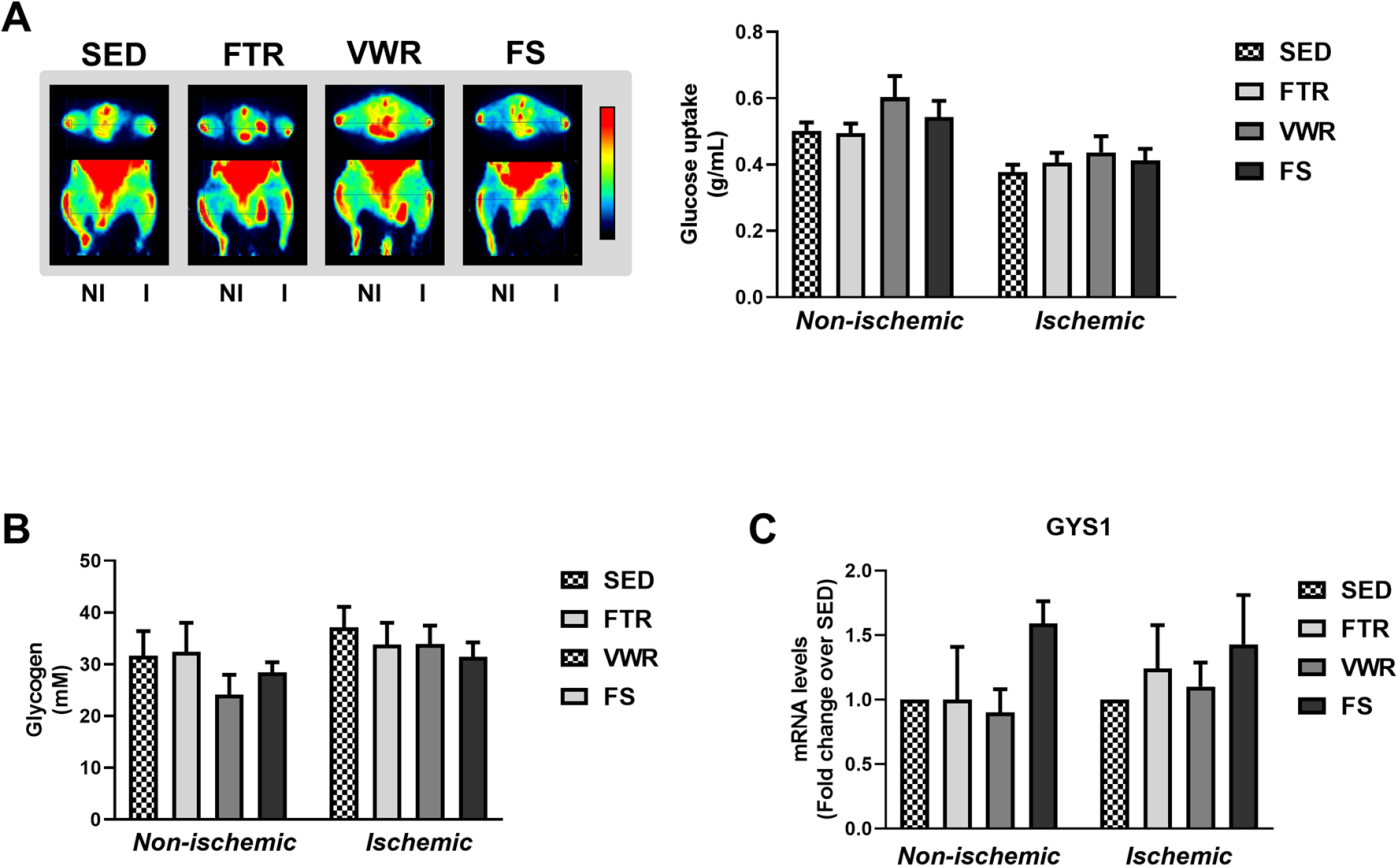
Effect of aerobic exercise training type on hindlimb muscle glucose uptake and glycogen content in ApoE^−/−^ mice with LEAD. **(A)** Left panel, Representative ^18^FDG PET images of phosphorylated ^18^FDG at steady-state in resting ischemic (I, right) and contralateral non-ischemic (NI, left) hindlimbs of exercised and SED mice at the study endpoint. The color scale ranges from low (black) to high (red) glucose uptake. Rectangles indicate regions of interest (ROI) over gastrocnemius hindlimb muscles. Right panel, Semi-quantification of hindlimb muscle glucose uptake, expressed in g/mL. Data represent mean ± SEM (n = 9 in SED; n = 6 in FTR, n = 11 in VWR, and n = 12 in FS). **(B)** Quantification of glycogen content from non-ischemic and ischemic gastrocnemius muscle, at the study endpoint. Data represent mean ± SEM (n = 6 in SED; n = 4 in FTR, n = 3 in VWR, and n = 6 in FS). **(C)** mRNA expression of GSY1 gene involved in glycogen synthesis in non-ischemic and ischemic gastrocnemius muscle at the study endpoint, as measured by quantitative real-time PCR. Results in the exercised groups are expressed as an x-fold change relative to SED hindlimb muscle, set at 1 (n = 6 mice per group). Data were analyzed using a one-way ANOVA with Dunnett’s post-hoc test.

Blood glucose and muscle glycogen are important fuels for increased adenosine triphosphate (ATP) production within contracting muscle during ET. To address the question whether the type of ET has a differential effect on muscle glucose metabolism, glucose uptake was determined at the study endpoint in contralateral and ischemic hindlimb muscles of individual mice comprising the SED, FTR, VWR and FS groups. Representative ^18^FDG PET images (Fig. 3A, left) and corresponding quantitation of glucose uptake (Fig. 3A, right) show no significant difference in glucose uptake by non-ischemic (NI) hindlimb muscle across all four groups (0.49 ± 0.03 g/mL, FTR; 0.60 ± 0.06 g/mL, VWR; 0.54 ± 0.05 g/mL, FS; 0.50 ± 0.03 g/mL, SED). Glucose uptake by ischemic hindlimb muscle, shown on the right of the same PET images, was lower than that of NI muscle, and comparable across all four groups (0.41 ± 0.03 g/mL, FTR; 0.44 ± 0.05 g/mL, VWR; 0.41 ± 0.04 g/mL, FS; 0.38 ± 0.03 g/mL, SED). This finding led us to ask whether the stored form of glucose, i.e. glycogen (Fig. 3B), or mRNA expression of glycogen synthase (GYS1, catalyzing the rate-determining step for muscle glycogen synthesis) (Fig. 3C) were differentially modulated in the hindlimb muscles. Figure 3B,C show that, muscle glycogen and mRNA expression of glycogen synthase did not differ between SED, FTR, VWR and FS groups, irrespective of muscle condition (non-ischemic vs. ischemic).

**Figure 4:**
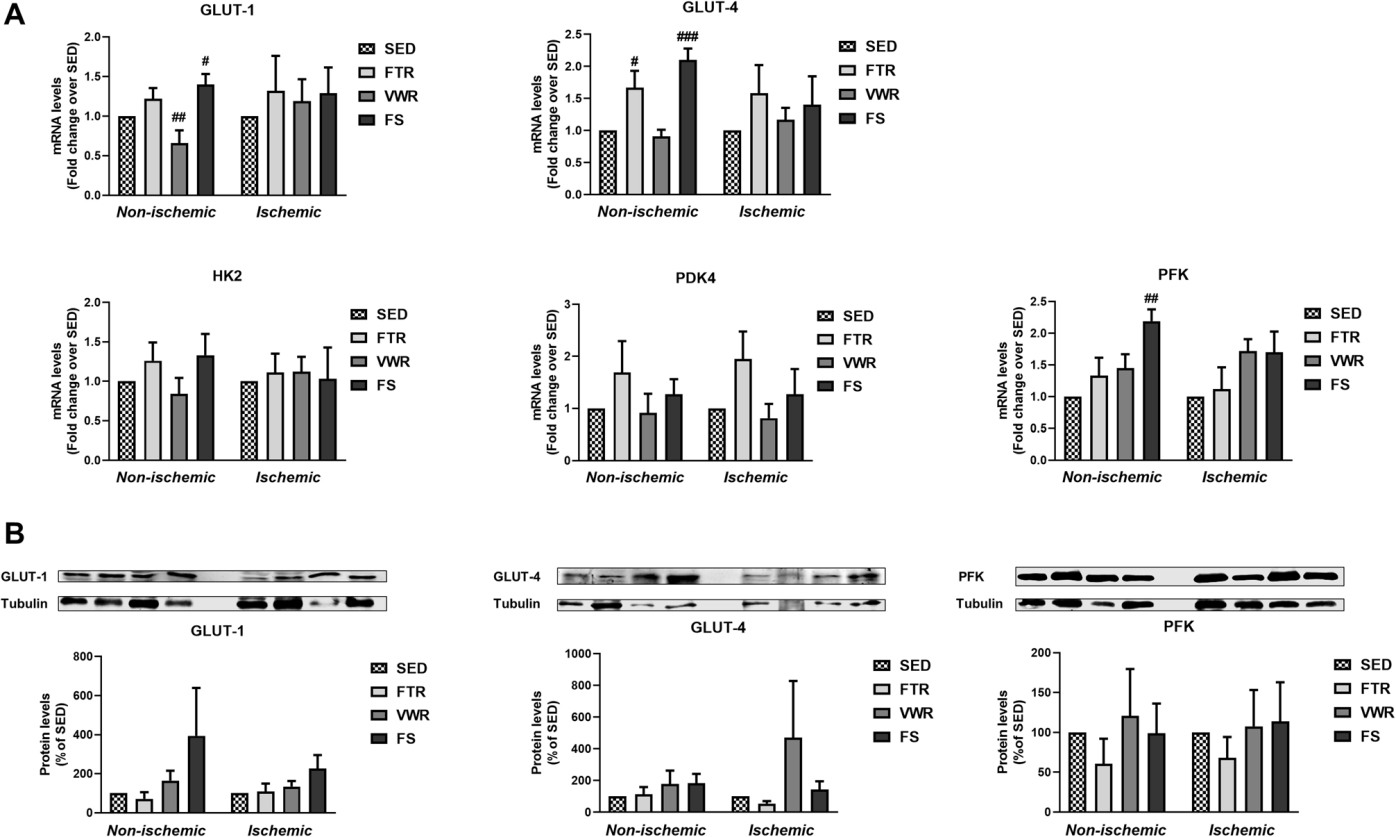
Effect of aerobic exercise training type on hindlimb muscle expression of genes associated with glucose metabolism in ApoE^−/−^ mice with LEAD.** (A)** mRNA expression of genes involved in glucose transport (GLUT-1 and GLUT-4) and glycolysis (HK2, PFK, and PDK4) in non-ischemic and ischemic gastrocnemius muscle at the study endpoint, as measured by quantitative real-time PCR. Results in the exercised groups are expressed as an x-fold change relative to SED hindlimb muscle, set at 1 (n = 6 mice per group). **(B)** Representative blots and quantitative analysis of GLUT-1, GLUT-4, and PFK protein expression levels in non-ischemic and ischemic gastrocnemius muscle at the study endpoint, as determined by Western blotting. Note that only the bands at the adequate molecular weights were shown here: tubulin (55 kDa), GLUT-1 (54 kDA), GLUT-4 (54 kDa) and PFK (85 kDa). Results in the exercised groups are expressed as percentage of SED, set at 100% (n = 4 mice per group). Data were analyzed using 1-way ANOVA with Dunnett’s post-hoc test (quantitative real-time PCR data) or Kruskal–Wallis with Dunn’s post-hoc test (Western blot data): ^#^P < 0.05, ^##^P < 0.01, ^###^P < 0.001 vs. SED.

Since glucose metabolism is also influenced by glucose transporters (GLUT-1, GLUT-4) and key regulatory enzymes in glycolysis, e.g. hexokinase (HK), phosphofructose kinase (PFK), and pyruvate dehydrogenase kinase (PDK), we examined their corresponding mRNA expression in non-ischemic and ischemic hindlimb muscle (Fig. 4A). In ischemic muscle, no significant difference was observed for GLUT-1, GLUT-4, HK2, PFK and PDK4 mRNA expression between the exercised and SED groups. In non-ischemic muscle, GLUT-1 mRNA expression significantly decreased by VWR (p < 0.01), whereas its expression significantly increased by FS mice (p < 0.05), when compared to the SED group (Fig. 4A). GLUT-4 mRNA expression in the non-ischemic muscle was significantly increased by FTR and FS (p < 0.05 and p < 0.001) when compared to the SEG group (Fig. 4A). PFK mRNA expression was significantly increased in the non-ischemic muscle of FS mice only (p < 0.05 vs. SED, Fig. 4A). However, western blot analysis revealed that the protein expression of GLUT-1, GLUT-4, and PFK did not significantly differ between the contralateral non-ischemic and ipsilateral ischemic hindlimb muscle across the exercised and the sedentary groups of mice (Fig. 4B).

These results indicate that exercise training has minimal effects on glucose transport and metabolism in ischemic hindlimb skeletal muscle.

### Fatty acid metabolism in hindlimb muscle following exercise training

**Figure 5:**
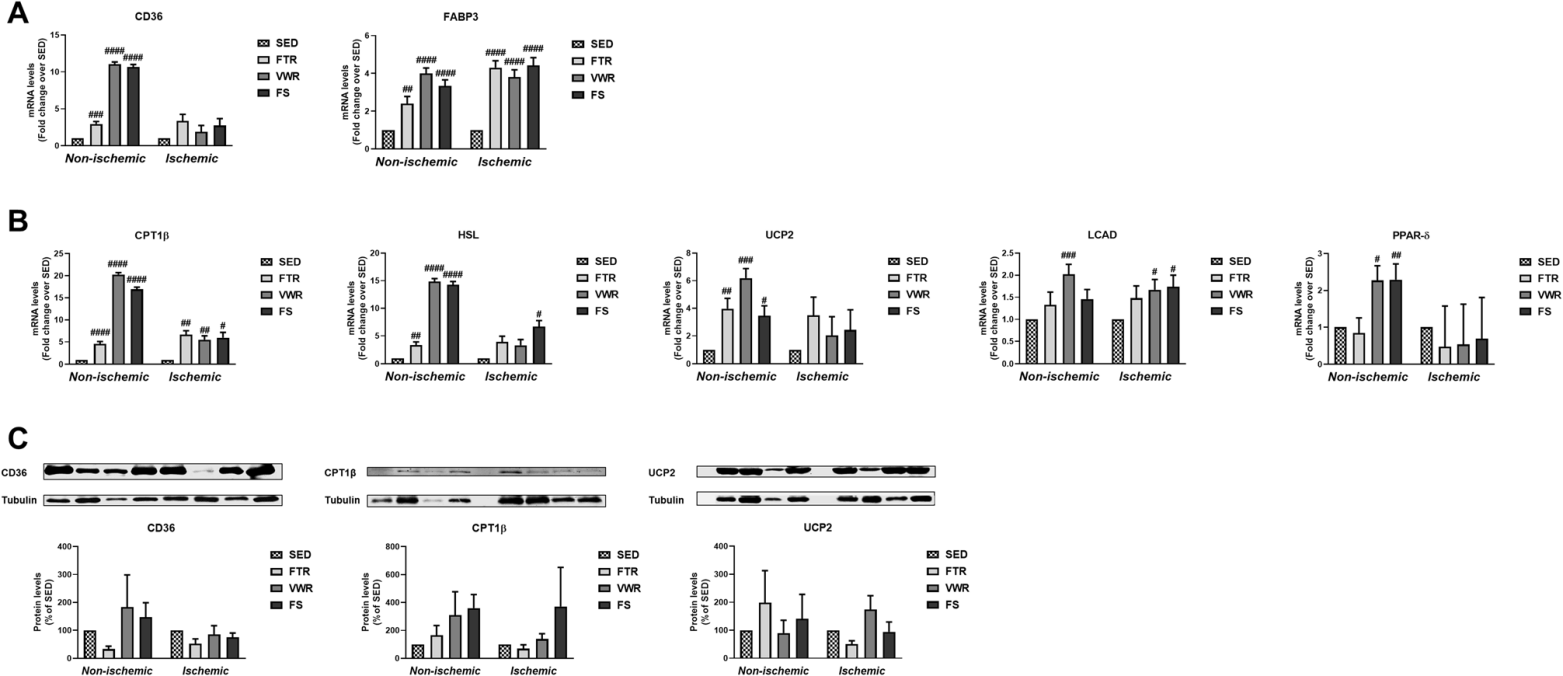
Effect of aerobic exercise training type on hindlimb muscle expression of genes associated with fatty acid metabolism in ApoE^−/−^ mice with LEAD. mRNA expression of genes involved in fatty acid uptake (CD36), transport (FABP3) **(A)**, and fatty acid β-oxidation (CPT1β, HSL, UCP2, LCAD and PPAR-δ) **(B)** in non-ischemic and ischemic gastrocnemius muscle at end of the study, as measured by quantitative real-time PCR. Results in the exercised groups are expressed as an x-fold change relative to SED mice, set at 1 (n = 6 mice per group). Representative blots and quantitative analysis of CD36, CPT1β, and UCP2 protein expression levels in non-ischemic and ischemic gastrocnemius muscle at the study endpoint, as determined by Western blotting **(C)**. Note that only the bands at the adequate molecular weights were shown here: tubulin (55 kDa), CD36 (88 kDA), CPT1β (88 kDa) and UCP2 (37 kDa). Results in the exercised groups are expressed as percentage of SED, set at 100% (n = 4 mice per group). Data were analyzed using one-way ANOVA with Dunnett’s post-hoc test (quantitative real-time PCR data) or Kruskal–Wallis with Dunn’s post-hoc test (Western blot data): ^#^P < 0.05, ^##^P < 0.01, ^###^P < 0.001, ^####^P < 0.0001 vs. SED.

Since skeletal muscle is able to use fatty acids as an energy substrate, in addition to glucose, we examined mRNA expression and the expression of protein products linked to fatty acid metabolism in hindlimb muscle following ET. The results are summarized in Fig. 5. In non-ischemic muscle, a significant increase was found in mRNA expression of five genes linked to either fatty acid uptake (CD36), transport (FABP3) or fatty acid β-oxidation (CPT1β, HSL, UCP2) by FTR, VWR and FS (p < 0.05 vs. SED). Increased mRNA expression of fatty acid β-oxidation genes LCAD and PPAR-δ occurred only in the non-ischemic muscle of VWR- and FS-trained mice. In ischemic hindlimb muscle, FABP3 and CPT1β mRNA expression was also significantly increased in the three exercise groups (p < 0.05 vs. SED, Fig. 5A, B). Ischemic muscle HSL mRNA expression was significantly increased in mice of the FS group only (p < 0.05 vs. SED), while LCAD mRNA expression significantly increased in VWR and FS groups (p < 0.05 vs. SED) (Fig. 5B). At the protein level, however there was no significant difference between the exercised and the SED groups for CD36, CPT1β, and UCP2 expression in the non-ischemic and ischemic muscle (Fig. 5C).

These results indicate that all types of exercise training stimulated mRNA expression of genes related to fatty acid metabolism, and that this increased expression was essentially confined to the non-ischemic muscle. However, the expression of key proteins associated with fatty acid metabolism was not increased in response to exercise training.

### Mitochondrial biogenesis and metabolism in hindlimb muscle following exercise training

**Figure 6:**
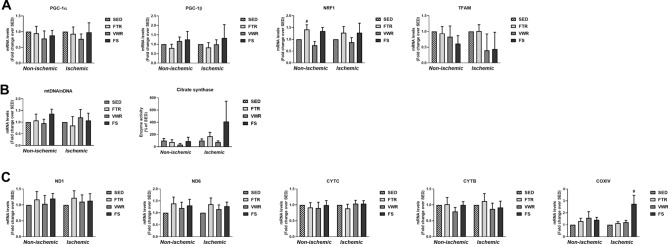
Effect of aerobic exercise training type on hindlimb muscle mitochondrial biogenesis and content in ApoE^-/-^ mice with LEAD. **(A)** mRNA expression of genes involved in mitochondrial biogenesis (PGC-1α, PGC-1β, NRF1, and TFAM), as measured by quantitative real-time PCR. **(B)** Relative mitochondrial DNA content, as measured by the analysis of the mtDNA/nDNA ratio, and citrate synthase activity. **(C)** mRNA expression of genes encoding mitochondrial respiratory chain complex (ND1, ND6, CYTC, COXIV, CYTB), as measured by quantitative real-time PCR. Results in the exercised groups are expressed as an x-fold change relative to non-ischemic or ischemic muscle of SED mice (quantitative real-time PCR data, n = 6–8 mice per group) or as percentage of SED mice (citrate synthase activity, n = 4 mice per group). Data were analyzed using one-way ANOVA with Dunnett’s post-hoc test: ^#^P < 0.05, ^##^P < 0.01, ^###^P < 0.001, ^####^P < 0.0001 vs. SED.

The final step of generating ATP from carbohydrates or from fatty acids necessitates the Krebs cycle and oxidative phosphorylation in the mitochondria. As shown in Fig. 6A, analysis of genes related to mitochondrial biogenesis revealed no significant change in mRNA expression of peroxisome proliferator-activated receptor γ coactivator 1α (PGC-1α), peroxisome proliferator-activated receptor γ coactivator 1β (PGC-1β) or transcription factor A (TFAM) between the different exercised groups in the non-ischemic and ischemic muscle. Only a significant increase was observed for nuclear respiratory factor 1(NRF1) mRNA expression in FTR-trained non-ischemic hindlimb muscle (p < 0.05 vs. SED, Fig. 6A). We then assessed mitochondrial DNA (mtDNA) copy number and citrate synthase activity as markers of mitochondrial quantity. As shown in Fig. 6A, mtDNA content and citrate synthase activity did not significantly differ between groups, whether the hindlimb was ischemic or non-ischemic. Finally, no significant changes in mRNA levels of key genes encoding subunits of the electron transport chain were found in response to ET, and this included mitochondrial NADH dehydrogenase 1 (ND1), mitochondrial NADH dehydrogenase 6 (ND6), mitochondrial cytochrome b (CYTB), cytochrome c (CYTC) (Fig. 6C). Of note, is a significant increase in cytochrome c oxidase subunit 4 (COXIV) mRNA expression, detected only in the ischemic hindlimb muscle of mice of the FS group (p < 0.05 vs. SED; Fig. 6C).

From these results, we concluded that exercise training is not associated with adaptive changes in mitochondrial content or mRNA levels of mitochondrial genes.

### Atherosclerosis progression

To examine whether ET type might influence the development of atherosclerosis, we performed a histological analysis of the aortic sinus. As expected, SED ApoE^−/−^ mice developed atherosclerotic lesions (Supplementary Fig. 2). Compared to SED mice, FTR mice showed significant (68%) lesion size reduction (p < 0.05). Neither VWR nor FS training prevented lesion development (Supplementary Fig. 2).

## Discussion

The major findings of this study were that (1) running, both forced and voluntary, improved voluntary exercise capacity, while forced swimming did not, in our LEAD mouse model; (2) relative to the sedentary group of mice, none of the ET paradigms enhanced ischemic hindlimb perfusion or oxygenation recovery, nor did ET increase vascularization or arteriogenic-related gene expression; (3) none of the ET paradigms increased glucose uptake, glycogen levels, or mRNA and protein expression of genes involved in glucose metabolism in non-ischemic and ischemic hindlimb muscle; (4) however, all modes of ET, however, increased mRNA (but not protein) expression of genes associated with lipid metabolism, particularly in ischemic muscle; and finally, (5) exercise training failed to stimulate mRNA expression of mitochondrial biogenesis-related genes, or modulate mitochondrial content in the non-ischemic and ischemic hindlimb muscle.

The first aim of the present study was to identify which exercise regime was effective in improving voluntary exercise capacity in our LEAD mouse model. In agreement with human studies^13,14^, we found that running was the most effective. Indeed, forced and voluntary running exercise were equally effective in improving 24-TRD, in spite of their differences: while the activity pattern of the running wheel is characterized by several short bouts (~ 150 s each) of high-speed running, separated by short breaks (~ 30 s), treadmill running forces the animal to run for a sustained period at moderate speed^15^. Although the total distance per day was relatively high with the voluntary running wheel (and much higher than the daily distance covered by mice engaged in forced treadmill running in our study), this exercise regimen resembles interval training in humans^15^. Of considerable interest, our findings in mice are in agreement with a recent pilot study in LEAD patients showing that high-intensity interval training therapy may be preferable to treadmill exercise^16^. Taken together, these results provide a rationale for conducting additional research with this type of ET in LEAD patients.

Swimming represents a potential, alternative mode to walking and running. We and others have previously shown that aerobic swimming slows atherosclerotic plaque development^17–19^ and protects against acute myocardial infarction^20^. Contrary to our working hypothesis, forced swimming did not have any beneficial effect on voluntary endurance exercise performance, at least in our LEAD mouse model. Further studies are however needed to determine whether swimming could be of clinical relevance in LEAD.

Whether hemodynamic changes can directly account for walking performance improvements in LEAD patients is still a matter of debate. Our experiments showed that ET does not trigger hindlimb perfusion and oxygenation, regardless of the ET paradigm. In fact, ischemic hindlimb perfusion and oxygenation increased over time in all groups of LEAD mice, including the sedentary ones. Moreover, histological and gene expression analysis showed that none of the ET protocols tested in our study stimulated ischemic muscle arteriogenesis. In agreement with our results, Nagase et al. ^21^ observed no change in hindlimb blood flow after four sessions of 30-min treadmill exercise in a mouse model of LEAD, 3 weeks after bilateral femoral artery ligation^21^. On the other hand, Cheng et al. ^22^ showed that 2 weeks of swimming (1 h per day) increased hindlimb perfusion of aged (18–24 months old) C57BL/6 mice in addition to increasing capillary density, numbers of collateral vessel, and protein levels of VEGF and HIF-1α in ischemic hindlimb 15 days after unilateral hindlimb ischemic surgery^22^. Moreover, Willmann et al. ^23^ reported increased ischemic muscle microvessel density and VEGF receptor 2 protein expression after 4 weeks of forced treadmill exercise in C57BL/6 mice with unilateral hindlimb ischemia^23^. Lastly, in mice with diet-induced obesity and unilateral femoral artery ligation, daily forced treadmill running for 4 weeks enhanced capillary density, but mRNA and protein levels of VEGF in the tibialis anterior muscle remained unchanged^24^. While these differences might be attributed, in part, to inter-laboratory differences in methodology and protocol, we have strived in our LEAD mouse study to identify which exercise regimen is effective at improving voluntary exercise capacity, and whether or not underlying mechanisms implicate hemodynamic and/or metabolic adaptations. To our knowledge, we show for the first time that improvement in voluntary endurance exercise performance following ET is not associated with enhanced blood flow, oxygenation and/or vascularization in a mouse model of LEAD. During the revision process of our manuscript, Krishna et al. ^25^ reported that 4 weeks of running wheel exercise (30–45 min per day,average distance covered per day: 80–200 m) increased functional capacity (i.e. total distance walked by mice until exhaustion) but not limb perfusion in ApoE^−/−^ mice subjected to a two-stage hindlimb ischemia (i.e. initial gradual femoral artery occlusion by ameroid constrictors for 14 days and subsequent excision of the femoral artery)^25^. These findings are directly in line with our observations. In accordance with our preclinical data, a systematic review of randomized controlled clinical trials including 1,237 LEAD subjects showed that exercise did not significantly change lower extremity hemodynamics in most trials, nor were clinical improvements related to changes in resting and post-exercise ankle brachial index (ABI, a cumulative measure of blood supply to the lower extremity) or reactive hyperemic calf blood flow^26^. Two recent systemic reviews and meta-analyses from Parmenter et al.^27^ and Lane et al.^4^ have confirmed that ET does not change ABI. Finally, Versluis et al.^28^ demonstrated an absence of collateral artery formation and flow increases subsequent to 3 or 6 months of supervised treadmill ET in IC patients, in spite of a significant improvement in pain-free and total walking distance^28^. Taken together, these data indicate that factors inherent in the muscle itself are most likely to play a role in exercise-induced clinical improvements in LEAD patients^10,11^.

During ET, working skeletal muscle requires increased energy substrates for the generation of ATP. Accordingly, metabolic pathways of ATP generation are active simultaneously, with carbohydrates and fat being the major energy substrate sources^29^. Unlike studies up to now, we addressed which mode of ET has the capacity to modulate glucose and/or fatty acid metabolism in non-ischemic and ischemic hindlimb muscles of LEAD mice.

Blood glucose is one source of energy for skeletal muscle during exercise. Indeed, Kemppainen et al.^30^ showed that chronic ET improved exercise capacity of patients with dilated cardiomyopathy with a concomitant enhancement in resting skeletal muscle glucose uptake, but without any changes in muscle blood flow and oxygen uptake^30^. However, in our study, FDG PET analyses revealed that 4 weeks of ET had no effect on control or ischemic hindlimb muscle glucose uptake. Along with this data, our study revealed that forced treadmill and voluntary wheel running have minimal effect on the expression of genes involved in glucose transport and utilization. Indeed, we observed only increases in GLUT-4 mRNA levels with forced treadmill running in the non-ischemic muscle. In line with this result, Burch et al.^31^ showed an increasing gene expression of GLUT-1 and GLUT-4 in non-ischemic gastrocnemius muscle and improved exercise performance in wild-type C57BL/6J mice following 6 weeks of treadmill training^31^. However, we observed herein an increase in mRNA expression of GLUT-4, but also of GLUT-1 and PFK (the rate-limiting enzyme of glycolysis) in response to forced swimming in the non-ischemic muscle, indicating that the increased GLUT-4 mRNA expression might not account for the benefit of forced running on endurance capacity. Importantly, we reported that ET did not increase muscle protein levels of these three genes (i.e., GLUT-1, GLUT-4 and PFK), indicating that most adaptation/regulation of muscle glucose metabolism in response to ET occurred at the transcriptional level (i.e. increased mRNA expression) rather than at the translational level in our mouse model. HK2 and PDK4 are key enzymes implicated in the control of glucose metabolism: HK2 phosphorylates glucose to glucose-6-phosphate before entering glycolysis or storage as glycogen, while PDK4 negatively regulates the pyruvate dehydrogenase complex, thus inhibiting the entry of pyruvate to the Krebs cycle. Here, the mRNA expression level of these two enzymes was not influenced by any type of ET in the ischemic and non-ischemic muscle. In support of our finding, Manio et al.^32^ reported no significant increase in gastrocnemius muscle PDK4 mRNA expression in C57BL/6 mice after 30 days of treadmill running^32^. One important finding of our study was that no change in glucose metabolism could be observed in ischemic-trained muscle. To our knowledge, our study is the first to demonstrate that no adaptation in glucose metabolism occurs in the ischemic hindlimb muscle in response to exercise training of LEAD mice.

Like blood glucose, intramuscular glycogen is a carbohydrate source for energy production for contracting skeletal muscle. Following exercise, glycogen synthase is activated and muscle glycogen is increased in the resting muscle^29^. In our study, ET did not modulate muscle glycogen, nor did it modulate the mRNA expression of GSY1. When taken together, our results show that improved endurance capacity in response to running is not due to an adaptation of carbohydrate metabolism in the skeletal muscle.

In addition to carbohydrates, another major source of energy for the working skeletal muscle during ET is fatty acids. Increased fat utilization could a priori contribute to improved endurance capacity in our running exercised LEAD mice. In order to gain insight about fatty acid metabolism in our exercised mice, we determined muscle expression levels of key proteins and enzymes involved in fatty acid uptake (FAT/CD36), transport (FABP3), and oxidation (CPT1β, HSL, UCP2, LCAD and PPAR-δ). In the non-ischemic muscle, each type of ET led to increased mRNA expression of all these genes (except for LCAD which was not up-regulated with forced treadmill running and swimming, and for PPAR-δ which was not up-regulated with forced treadmill running), indicating an activation of the different steps of the fatty acid metabolic pathway at the transcriptional level. An increased mRNA expression of CD36, FABP3, CPT1β, UCP2, HSL and PPAR-δ has also been reported in trained non-ischemic gastrocnemius mouse muscle^32,33^. Of note, in the ischemic muscle, transcriptional adaptations indeed occur but to a lesser extent. We also determined the expression of some proteins for which increased gene expression was observed in exercised groups. We found no increased protein levels for CD36, CPT1β and UCP2 in response to ET. Overall, the fact that fatty acid metabolism-related gene expression was stimulated in response to all three types of ET (i.e. FTR, VWR and FS) and that these changes did not occur at the translational level suggests that this metabolic adaptation is not likely to underlie improvements in endurance capacity of FTR and VWR exercised mice.

It is well known that aerobic ET elicits intramuscular adaptations, such as increases in mitochondrial biogenesis, oxidative capacity, and mitochondrial density. Mitochondrial biogenesis is regulated by the activation of the PGC-1α. This master regulator of mitochondrial biogenesis activates NRF-1, NRF-2 and TFAM, which enables the expansion of mitochondrial size and transcription of mitochondrial DNA^34^. Given that increasing mitochondrial biogenesis and content represents a key mechanism for the improvement in endurance performance following training, we hypothesized that running-exercised LEAD mice would have a greater expression of mitochondrial genes and increased mitochondrial content in the non-ischemic muscle, as previously reported after chronic aerobic treadmill or voluntary running exercise in C57BL/6 mice^31,32,35,36^, but also in the ischemic muscle. Contrary to our expectations, we did not observe any effect of ET on mitochondrial biogenesis markers (mitochondrial DNA content, citrate synthase activity, and mRNA expression of transcriptional regulators and genes encoding mitochondrial respiratory chain complex), in the non-ischemic and ischemic muscle. In a critical limb ischemia murine model (i.e. the advanced stage of LEAD), Lejay et al.^37^ reported that moderate forced treadmill running for 3 weeks induced increased transcript levels of PGC-1α, PGC-1β and NRF-1 in both non-ischemic and non-ischemic tibialis muscle^37^. In LEAD mice, results are inconsistent with the study from Albadawi et al.^24^ showing upregulation of ischemic tibialis muscle PGC-1α mRNA level^24^, and the study of Nagase et al.^21^, which like ours observed no increase in ischemic soleus muscle PGC-1α mRNA expression^21^ after treadmill exercise. Again, none of these animal studies have examined whether mitochondrial content and expression of genes involved in mitochondrial-related biogenesis can be influenced by exercise type, as well as whether such possible adaptations translate into a functional gain in hindlimb use. In IC patients that performed 8 weeks of unsupervised calf muscle exercise that led to an improved walking performance, van Schaardenburgh et al.^38^ showed an increase in mitochondrial content in the gastrocnemius muscle^38^. On the other hand, the same study reported no change in mitochondrial content in another group of IC patients that performed 8 weeks of unsupervised walking, but with no gain in walking performance^38^. Additional studies are needed to further clarify the role of mitochondrial function in mediating the beneficial effects of ET in LEAD.

LEAD is a clinical manifestation of systemic atherosclerosis; most patients with LEAD have clinically significant coronary artery disease and cerebrovascular disease. Although the primary goal of the present study was not to examine the effect of ET on atherosclerosis, our study is the first one to our knowledge to directly compare the effect of forced treadmill running, voluntary wheel running, and forced swimming on early atherosclerosis development. We showed that forced treadmill walking, but not voluntary wheel running or forced swimming, prevented early atherosclerosis progression. A significant reduction in aortic lesion size has been recently reported in young ApoE^−/−^ mice (without LEAD) after 5 weeks of a treadmill endurance program^39^. Our data emphasizes the clinical relevance of endurance treadmill ET for reducing cardiovascular mortality in LEAD patients as previously reported^40^.

In conclusion, we report that forced and voluntary running have similar beneficial effects on voluntary exercise capacity. Forced swimming had no effect, at least in our mouse model of LEAD. In addition, this study demonstrated that neither running nor swimming enhance vascularization and blood flow to the ischemic limb, in line with little or no changes in glucose metabolism and mitochondrial biogenesis markers in the skeletal muscle. All types of exercise increased the mRNA expression (but not the protein expression) of genes encoding fat metabolism in skeletal muscle, an indication that this metabolic adaptation does not account for running exercise-induced voluntary endurance exercise performance improvement. These observations challenge the existing hypothesis and highlight the multifaceted adaptations regarding the mechanisms underlying the benefits of exercise training in LEAD.

## Methods

Unilateral (right) hindlimb ischemia was induced in 11 to 16-week old male hypercholesterolemic and atherosclerotic C57BL/6 Apolipoprotein E knock-out (ApoE^−/−^) mice by surgical ligation of the right common iliac artery as previously described^12^. Under isoflurane anesthesia, this procedure necessitated only a small abdominal incision, before the right common iliac artery was exposed and ligated.

Mice were housed at the Animal Care Facility at the University Hospital of Lausanne (Lausanne, Switzerland) under conventional conditions with free access to standard rodent chow and water. Animal experiments were carried out in accordance with Swiss Federal Veterinary Regulations and were approved by the local committee (Service of Consumption and Veterinary Affairs, Canton of Vaud).

### Experimental groups and exercise protocols

One week after surgery (i.e. baseline), mice were randomly allocated to one of four groups: (1) forced treadmill running (FTR); (2) voluntary wheel running (VWR); (3) forced swimming (FS); and (4) sedentary control group (SED).

Animals were euthanized, and gastrocnemius muscles collected at the study endpoint (i.e. 5 weeks after surgery or 4 weeks after exercise commencement), and at least 24 h after their final exercise session to avoid any acute effects of exercise.

#### Forced treadmill running protocol

The treadmill exercise program was adapted from a previously published one^23^. Mice ran on a motor treadmill (Columbus Instruments, Columbus OH, USA) 5 days/week. Each training session started at a speed of 9 m/min for 3 min with an increase of 2 m/min every 3 min until a maximum speed of 19 m/min was reached (0% slope). Mice were encouraged to run with the use of an electric grid located at the back of the treadmill. Training was stopped when mice remained on the electric grid for 5 continuous seconds. Mice were acclimated to the treadmill 1 week before surgery (5 m/min for 10 min).

#### Voluntary wheel running protocol

Mice were housed individually in a cage supplied with a 12 cm diameter wheel and were free to run 7 days/week. The wheel was connected to a counter, recording the daily number of revolutions, allowing for the calculation of running distance.

#### Forced swimming protocol

Mice swam 60 min/day, 5 days/week in water maintained at 35 °C to 36 °C as previously described^19^. Mice were progressively trained during the first week after surgery: 10 min swimming on day 1, followed by daily 10-min increases up to 50 min swimming on day 5. Following this first week, swimming was set to 60 min per day. After swimming sessions, wet animals are carefully dried.

### Voluntary exercise capacity assessment

To assess voluntary endurance exercise performance of mice, a wheel running test was used to assess the total running distance (in km) covered over a 24-h period, abbreviated as 24 h-TRD. 24 h-TRD was evaluated at 3 time points: before surgery, 1 week post-surgery (i.e. baseline), and 5 weeks post-surgery (i.e. study endpoint).

### Laser Doppler perfusion imaging of hindlimb

Tissue perfusion of the ischemic and contralateral non-ischemic hindlimbs was evaluated at rest before surgery, at baseline, and at end of the study using a laser Doppler Imager (Moor Instruments, Axminster, UK) in anesthetized mice as described previously^12^.

### Transcutaneous partial pressure of oxygen (TcPO_2_) measurement in hindlimb

Resting tissue oxygenation of ischemic hindlimbs was determined by measuring the transcutaneous partial pressure of oxygen using a TcPO_2_-monitoring system equipped with a Clark electrode (TCM30; Radiometer, Copenhagen, Denmark) as previously detailed^12^. TcPO_2_ measurements were performed before surgery, at baseline, and at end of the study.

### ^18^FDG Positron emission tomography of hindlimb glucose metabolism

Non-invasive, in vivo measurement of glucose uptake by hindlimb muscle at rest was performed with positron emission tomography (PET) as previously described in detail^12,41^. Briefly, mice were first scanned at baseline and then rescanned 1 month later at the end of the study. Catheter insertion of the tail vein allowed for ^18^FDG substrate administration, and blood sampling to establish initial and final glycemia. Mice were prone positioned on the scanner bed to accommodate continued delivery of isoflurane via a nose mask, reproducible positioning of extended lower limbs within a single field of view, and continuous monitoring of physiology. Fifty-minute list mode acquisitions were acquired with the field of view (FOV) centered on the hindlimbs and initiated with i.v. injection of ^18^F-fluorodeoxyglucose (^18^FDG) (∼ 50 MBq) through the tail vein catheter within the first 10 s of the PET scan, followed by 100–300 μL of saline chase solution. Imaging was performed using an avalanche photodiode, dedicated small animal micro-PET scanner (LabPET4; Gamma Medica, Sherbrook, Canada). During the entire scanning period, mice were maintained under 1% (vol/vol) isoflurane anesthesia in oxygen using a face mask with constant monitoring of temperature and breathing rate. List mode data were sorted into 1 min time frames of which the last 20 min of the 50 min scan was used to determine the standardized uptake value (SUV), defined as (mean ROI activity [kBq/cm^3^])/(injected dose [kBq]/body weight [g]), providing a measure of accumulated intracellular ^18^FDG-6-phosphate at steady-state. Voxel size measured 0.5 × 0.5 × 1.2 mm, giving a typical resolution of 1.2 mm at the center of the FOV. Regions of interest (ROI) were drawn manually by optical reading of well-delineated hindlimb muscle during image analysis with PMOD software (Version 3.2; PMOD Technologies).

### Enzymatic bioassay of glycogen in hindlimb muscle

At the end of the study, 40 mg on average of fresh gastrocnemius muscle was excised from both ischemic and non-ischemic hindlimbs from FTR, VWR, FS and SED mice, and then plunged and stored in liquid nitrogen until biochemical measurements. The procedure for the assay is based on the enzymatic release of glucose from glycogen by amyloglucosidase according to the protocol of Cruz and Dienel (referenced in^42^, glucose oxidase digestion, and spectrophotometric absorbance detection of glucose in the form of resorufin following the protocol of Poitry-Yamate et al.^42^.

### Histological and immunohistochemistry analyses

At the end of study, gastrocnemius muscles of the right and left hindlimbs were isolated and fixed with 10% buffered formalin. After fixation, specimens were paraffin embedded, and tissue cross-sections (5 µm thick) were prepared. Muscle fiber size (μm^2^) was determined from hematoxylin and eosin stained sections. For each sample, a minimum of 30 muscle fibers sizes were quantified in five randomly selected fields, and the results averaged. Stained sections were also used for assessment of muscle fiber regeneration, i.e. the number of fibers with central nuclei. The number of regenerated fibers was expressed as % of total number of muscle fibers.

For vascularization assessment, muscle sections were immunostained with a mouse monoclonal α-smooth muscle actin (α-SMA) antibody, followed by a secondary biotinylated anti-mouse antibody^12^. Arteriolar density was defined as the number of arterioles per muscle fiber or per high power field in ischemic and non-ischemic hindlimb muscle. All morphometric analyses were performed using Qwin analysis software (Leica).

### Real-time reverse transcription-polymerase chain reaction analysis

Total RNA was isolated from frozen ischemic and contralateral non-ischemic gastrocnemius muscles using Trizol reagent (Invitrogen, Switzerland), followed by the RNeasy Cleanup Kit (Qiagen, Switzerland), or using RNeasy Fibrous Tissue Mini Kit (Qiagen, Switzerland) according to the manufacturer’s protocol. Quantitative real-time PCR was performed on a CFX96 Real-Time PCR detection system (Bio-rad, Switzerland) as previously published^12^. Primers were designed using the NCBI-primer-BLAST webserver and were synthesized by Microsynth (Switzerland). The primer sequences are listed in Supplementary Table 2. Data were analyzed using the comparative threshold cycles (CT) method with 36B4 gene as internal control for normalization, and relative gene expression was calculated using the 2^−ΔΔCT^ formula.

### Western blotting

Total proteins were extracted from non-ischemic and ischemic gastrocnemius muscles and transferred to nitrocellulose membranes before staining with primary antibodies overnight at 4 °C. For this purpose, tissue powder was lysed in buffer containing Nonidet P-40 0.5%, NaCl 150 mM, Na-orthovanadate 1 mM, NaF 10 mM, Tris–HCL pH = 7.5 10 mM, PMSF 1 mM, EDTA 1 mM (pH = 8), aprotinin 10 μg/ml, leupeptin 10 μg/ml, pepstatin 1 μg/ml, and centrifuged 30 min at 16 000 rpm. The amount of proteins was quantified by BCA assay and 20 μg of total protein were separated by SDS-PAGE, transferred to nitrocellulose membranes (Biorad), and blocked for 1 h at room temperature in Odyssey blocking buffer (1/2 in TBS Li-COR Biosciences). Membranes were incubated overnight at 4 °C with primary antibodies, such as rabbit anti-VEGFA (1/500, ab46154, Abcam), rabbit anti-GLUT-1 (1/1,000, ab652, Abcam), rabbit anti-GLUT-4 (1/3,000, ab654, Abcam), rabbit anti-PFKM (1/10,000, ab154804, Abcam), rabbit anti-CD36 (1/2000, ab133625, Abcam), rabbit anti-CPT1β (1/500, NBP1-59576, Novus Biologicals), goat anti-UCP2 (1/1,000 NB100-59742, Novus Biologicals) together with a mouse anti-tubulin (1/20,000, Sigma). Secondary antibodies, goat anti-rabbit coupled to Alexa 680 (1/5 000, Molecular Probes) or rabbit anti-goat IRDye700 (1/5,000, Rockland) or anti-mouse IRDye 800 (1/20,000, Rockland) were added 2 h at room temperature. The immunoblot signals were detected and quantified using the Odyssey infrared imaging system (LI-COR Biosciences, Bad Homburg, Germany). All results were corrected for their expression of tubulin.

### Mitochondrial DNA quantification

Total DNA was isolated from non-ischemic and ischemic gastrocnemius muscles using the DNAeasy blood and tissue kit (Qiagen, Switzerland). Quantification of mtDNA copy number was done by determining the mtDNA to nDNA ratio using quantitative real-time PCR as described above with specific primers according to the protocol of Quiros et al.^43^.

### Citrate synthase activity

Citrate synthase activity was determined in homogenates from non-ischemic and ischemic gastrocnemius muscles by using a commercial citrate synthase activity assay kit following the manufacturer’s instructions (CS0720, Sigma-Aldrich). In brief, enzyme activity was measured in a 190 µl reaction mixture in a 96-well plate containing 10 µg of total protein, an assay buffer, 30 mM Acetyl CoA solution and 10 mM DTNB solution. Endogenous and total activities were calculated by measuring absorbance at 412 nm every 10 s for 1.5 min at 25 °C before and after addition of 10 μL oxaloacetate to initiate the reaction, respectively. All measurements were performed in duplicate on a Hidex sense microplate reader (Hidex).

### Atherosclerosis quantification

The extent of atherosclerosis was quantified in cross sections of the aortic sinus as previously described^19^. Briefly, hearts were dissected, fixed in 10% buffered formalin, and subsequently paraffin embedded. Hearts were serially cut into 3 μm-thick sections until reaching the aortic sinus, and then processed for Movats pentachrome staining. Plaque area was quantified using the Qwin software, and expressed in μm^2^.

### Total plasma cholesterol level measurements

At the end of the study, plasma cholesterol concentrations were determined spectrophotometrically using a commercial kit (Diasys Diagnostic systems GmbH, Holzhein, Germany).

### Statistical analysis

All data are expressed as mean ± SEM unless otherwise specified. Two-way repeated measures ANOVA was used to compare endurance capacity, hindlimb perfusion, and oxygenation data. One-way repeated measures ANOVA was used to compare ET protocols. Kruskal–Wallis was used to compare western blot data. One-way ANOVA was used to analyze other data. The Bonferroni’s, Dunnett’s, or Dunn’s tests were applied for multiple comparisons among the experimental groups. A value of p < 0.05 was considered statistically significant. Analyses were performed using GraphPad Prism (GraphPad Software, Inc.).

## Supplementary information

Supplementary Table 1.

Supplementary Figure 1.

Supplementary Figure 2.

Supplementary Table 2.
