# Supplementary material for: Impact of aerobic exercise type on blood flow, muscle energy metabolism, and mitochondrial biogenesis in experimental lower extremity artery disease
Source: Sci Rep. 2020 Aug 20;10:14048. doi: 10.1038/s41598-020-70961-8 (PMC7441153; doi:10.1038/s41598-020-70961-8)
Supplement: Supplementary file 1 — Supplementary Table 1. [file 41598_2020_70961_MOESM1_ESM.pdf]

Impact of aerobic exercise type on blood flow, muscle energy metabolism, and mitochondrial biogenesis in experimental lower extremity artery disease

Maxime Pellegrin[1]*, Karima Bouzourène[1], Jean-François Aubert[1], Christelle Bielmann[1], Rolf Gruetter[2], Nathalie Rosenblatt-Velin[1], Carole Poitry-Yamate[2], Lucia Mazzolai[1]

[1]Division of Angiology, Heart and Vessel Department, University Hospital of Lausanne (CHUV), Lausanne, Switzerland

[2]Center for Biomedical Imaging (CIBM), Ecole Polytechnique Fédérale de Lausanne (EPFL), Lausanne, Switzerland

ADDITIONAL INFORMATION

Supplementary Table 1. Total weekly running distance (km) covered by individual ApoE$^{-/-}$ mice with LEAD.

Group	First week of training	Second week of training	Third week of training	Fourth week of training
FTR	0.98 ± 0.10	1.16 ± 0.05	1.46 ± 0.04$^{\&,££}$	1.49 ± 0.08$^{\&,££}$
VWR	3.84 ± 0.39	6.63 ± 0.22$^{\&\&\&}$	7.44 ± 0.24$^{\&\&}$	6.66 ± 0.43

Data are mean ± SEM (n=19 animals per group).

Data were analyzed using one-way repeated measures ANOVA with Bonferroni's post-hoc test: $^{\&}$P<0.05, $^{\&\&}$P<0.01, $^{\&\&\&}$P<0.001, vs. first week of ET; $^{££}$P<0.01 vs. second week of ET.
