# Supplementary material for: Impact of aerobic exercise type on blood flow, muscle energy metabolism, and mitochondrial biogenesis in experimental lower extremity artery disease
Source: Sci Rep. 2020 Aug 20;10:14048. doi: 10.1038/s41598-020-70961-8 (PMC7441153; doi:10.1038/s41598-020-70961-8)

Impact of aerobic exercise type on blood flow, muscle energy metabolism, and mitochondrial biogenesis in experimental lower extremity artery disease

Maxime Pellegrin[1]*, Karima Bouzourène[1], Jean-François Aubert[1], Christelle Bielmann[1], Rolf Gruetter[2], Nathalie Rosenblatt-Velin[1], Carole Poitry-Yamate[2], Lucia Mazzolai[1]

[1]Division of Angiology, Heart and Vessel Department, University Hospital of Lausanne (CHUV), Lausanne, Switzerland

[2]Center for Biomedical Imaging (CIBM), Ecole Polytechnique Fédérale de Lausanne (EPFL), Lausanne, Switzerland

Supplementary Figure 1. Effect of exercise training on arteriolar density in non-ischemic gastrocnemius muscle in ApoE$^{-/-}$ mice with LEAD.

Quantification of arteriolar density in non-ischemic gastrocnemius muscle at the study endpoint, expressed as the number of α-SMA-positive arterioles per muscle fiber and per high power field. Data represent mean ± SEM (n=5 per group).

Data were analyzed using two-way repeated measures ANOVA with with Dunnett's post-hoc test.

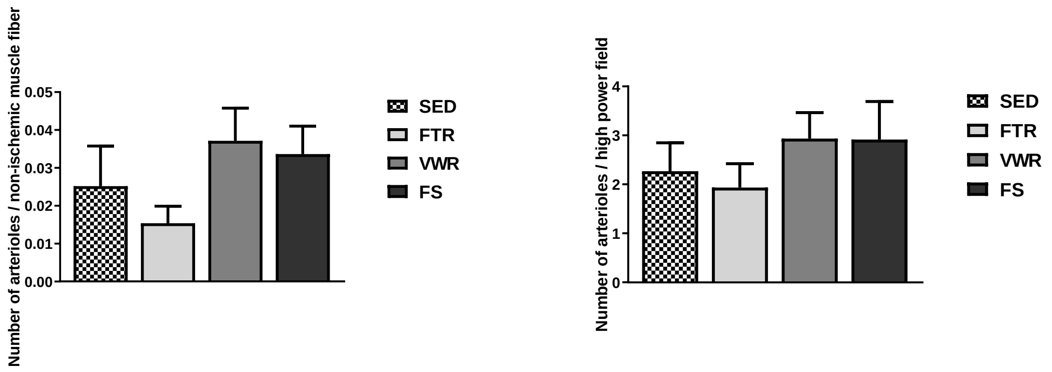

Supplement: Supplementary file 2 — Supplementary Figure 1. [file 41598_2020_70961_MOESM2_ESM.pdf]
