# Supplementary material for: Impact of aerobic exercise type on blood flow, muscle energy metabolism, and mitochondrial biogenesis in experimental lower extremity artery disease
Source: Sci Rep. 2020 Aug 20;10:14048. doi: 10.1038/s41598-020-70961-8 (PMC7441153; doi:10.1038/s41598-020-70961-8)
Supplement: Supplementary file 3 — Supplementary Figure 2. [file 41598_2020_70961_MOESM3_ESM.pdf]

Impact of aerobic exercise type on blood flow, muscle energy metabolism, and mitochondrial biogenesis in experimental lower extremity artery disease

Maxime Pellegrin[1]*, Karima Bouzourène[1], Jean-François Aubert[1], Christelle Bielmann[1], Rolf Gruetter[2], Nathalie Rosenblatt-Velin[1], Carole Poitry-Yamate[2], Lucia Mazzolai[1]

[1]Division of Angiology, Heart and Vessel Department, University Hospital of Lausanne (CHUV), Lausanne, Switzerland

[2]Center for Biomedical Imaging (CIBM), Ecole Polytechnique Fédérale de Lausanne (EPFL), Lausanne, Switzerland

ADDITIONAL INFORMATION

Supplementary Figure 2. Effect of exercise training on atherosclerosis extension of ApoE$^{-/-}$ mice with LEAD.

A) Representative images of Movat's Pentachrome-stained cross-sections of aortic sinus from control and exercised mice after exercise protocols. The red crosses indicate the presence of atherosclerotic lesions. **B)** Quantification of lesion area expressed in μm^2.

Data represent mean ± SEM (n=8 in SED; n=7 in FTR, n=7 in VWR, and n=7 in FS).

Data were analyzed using one-way repeated measures ANOVA with Bonferroni's post-hoc test: $^{\#}P<0.05$ vs SED.

A

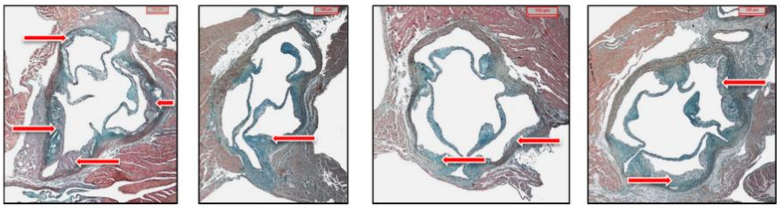

B

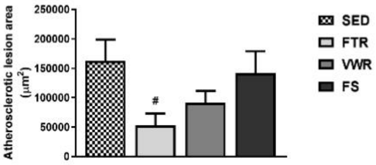
Atherosclerotic lesion area (μm²)
250000
200000
150000
100000
50000
0
#
SED
FTR
VWR
FS
