# Supplementary material for: Impact of aerobic exercise type on blood flow, muscle energy metabolism, and mitochondrial biogenesis in experimental lower extremity artery disease
Source: Sci Rep. 2020 Aug 20;10:14048. doi: 10.1038/s41598-020-70961-8 (PMC7441153; doi:10.1038/s41598-020-70961-8)
Supplement: Supplementary file 4 — Supplementary Table 2. [file 41598_2020_70961_MOESM4_ESM.pdf]

Impact of aerobic exercise type on blood flow, muscle energy metabolism, and mitochondrial biogenesis in experimental lower extremity artery disease

Maxime Pellegrin[1]*, Karima Bouzourène[1], Jean-François Aubert[1], Christelle Bielmann[1], Rolf Gruetter[2], Nathalie Rosenblatt-Velin[1], Carole Poitry-Yamate[2], Lucia Mazzolai[1]

[1]Division of Angiology, Heart and Vessel Department, University Hospital of Lausanne (CHUV), Lausanne, Switzerland

[2]Center for Biomedical Imaging (CIBM), Ecole Polytechnique Fédérale de Lausanne (EPFL), Lausanne, Switzerland

Supplementary Table 2. Mouse primer sequences used for quantitative real-time PCR.

Gene	Forward sequence	Reverse sequence	GenBank accession number	Product size (bp)
36B4	5’-ATGGGTACAAGCGCGTCCTG-3’	5’GCCTTGACCTTTTCAGTAAG-3’	NM_007475	72
VEGFA	5’-TGCACCCACGACAGAAGG-3’	5’-GCACACAGGACGGCTTGA-3’	NM_009505	155
HIF-1α	5’-TCAAGTCAGCAACGTGGAAG -3’	5’-TATCGAGGCTGTGTCGACTG-3’	NM_010431	197

ANGPT2	5’-GCATGTGGTCCTTCCAACTT -3’	5’-TGGTGTCTCTCAGTGCCTTG-3’	NM_007426	215
GYS1	5’-GAGAACGCAGTGCTTTTCGA-3’	5’-TCATCCCCTGTCACCTTCG-3’	NM_030678	97
GLUT-1	5’-GTGTATCCTGTTGCCTTC-3’	5’-GCTTCTTCAGCACACTCTT-3’	NM_011400	91
GLUT-4	5’-CTCTCAGGCATCAATGCTGTTTTCTA-3’	5’-CGAGACCAACGTGAAGACCGTATT-3’	NM_009204	184
HK2	5’-GCTAGGAGCTACCACACACCCT-3’	5’-ACTCGCCATGTTCTGTCCCATCC-3’	NM_013820	88
PFK	5’-GGAGTGCGTGCAGGTGACCAAA-3’	5’-ATCACGGCCACTGTGTGCAACC-3’	NM_001163487	170
PDK4	5’-TACTCCACTGCTCCAACACCTG-3’	5’-AGCCATAACCAAAACCAGCCAAAG-3’	NM_013743	69
CD36	5’-ATGGGCTGTGATCGGAACTG-3’	5’-GTCTTCCCAATAAGCATGTCTCC-3’	XM_006535621	109
FABP3	5’-ACCTGGAAGCTAGTGGACAG -3’	5’-TGATGGTAGTAGGCTTGGTCAT-3’	NM_010174	86
CPT1β	5’-TGGACCGTGAAGAGATCAAGC-3’	5’-CTCTTTGCCTGGGATGCGT-3’	NM_009948	103
HSL	5’-ACTGAGATTGAGGTGCTGTC-3’	5’-TGAGATGGTAACTGTGAGCC-3’	NM_010719	134
UCP2	5’-TTCCCTGTTGATGTGGTCAA-3’	5’-CAGTGACCTGCGCTGTGGTA-3’	NM_011671	70

LCAD	5’-CATATTCCCCCAGGACATTG-3’	5’-CACAATTGCCTCTATGTGCATT-3’	NM_007381	124
PPAR-δ	5’-ACCACTCGCATTCCTTTGAC-3’	5’-TGGGTCAGCTCTTGTGAATG-3’	XM_006505737	100
PGC-1α	5’- ACTATGAATCAAGCCACTACAGAC -3’	5’- TTCATCCCTCTTGAGCCTTTCG -3’	NM_008904	143
PGC-1β	5’-GAGGAGTCCCTTCCTTCATC-3’	5’-TCCTCGAAGGTTAAGGCTGA-3’	NM_133249	111
NRF1	5’-GCACCTTTGGAGAATGTGGT-3’	5’-CTGAGCCTGGGTCATTTTGT-3’	NM_001164226	165
TFAM	5’-CCAAAAAGACCTCGTTCAGC-3’	5’-CTTCAGCCATCTGCTCTTCC-3’	NM_009360	211
ND1	5’-GCACCTACCCTATCACTCACA-3’	5’-GTTTGGGCTACGGCTCG-3’	ENSMUSG00000064341	180
ND6	5’-TACCCGCAAACAAAGATCACC-3’	5’-ATGTTGGAAGGAGGGATTGGG-3’	ENSMUSG00000064368	87
CYTC	5’-CCAAATCTCCACGTTCTGTT-3’	5’-GTCTGCCCTTTCTCCCTTCT-3’	NM_007808	191
CYTB	5’-ACGCCATTCTACGCTCAATC-3’	5’-GCTTCGTTGCTTTGAGGTAT-3’	NC_005089	110
COXIV	5’-GCCTTGGACGGCGGAAT-3’	5’-CCACATCAGGCAAGGGGTAG-3’	NM_001293559	162
